# A Case Report on a Life-Threatening Occult Supraglottic Granulating Mass Masquerading as Life-Threatening Asthma and Presenting as Cardiac Arrest

**DOI:** 10.7759/cureus.117504

**Published:** 2026-10-05

**Authors:** Dimple Shamnani, Yun Hung Chor

**Affiliations:** 1 Medicine, University Hospitals Birmingham NHS Foundation Trust, Birmingham, GBR; 2 Emergency Department, Birmingham Heartlands Hospital, Birmingham, GBR

**Keywords:** acute respiratory failure, asthma, ball-valve obstruction, difficult airway, granulation tissue, pea cardiac arrest, post-extubation stridor, supraglottic mass, upper airway obstruction, video laryngoscopy

## Abstract

Structural upper-airway lesions are uncommon but important mimics of asthma. A 74-year-old man with asthma and multiple recent admissions for presumed exacerbations presented with acute dyspnoea and subsequently sustained a pulseless electrical activity (PEA) cardiac arrest. Return of spontaneous circulation was achieved after five minutes, followed by emergency intubation and admission to intensive care. He was diagnosed and treated for presumed acute severe asthma and respiratory infection.

By the following day, there was no significant wheeze or bronchospasm and extubation was attempted. He immediately developed severe stridor and profound hypoxaemia, with oxygen saturation falling to approximately 60%. Emergency video laryngoscopy revealed a large mobile supraglottic mass causing apparent “ball-valve” obstruction. Re-intubation restored oxygen saturation to 100%. The lesion was subsequently completely excised by the otorhinolaryngology team, with histopathology demonstrating granulation tissue.

This case highlights the importance of considering structural upper-airway pathology when presumed asthma follows an atypical course, particularly when post-extubation stridor persists despite resolution of bronchospasm.

## Introduction

Asthma is a common chronic respiratory disorder characterised by variable respiratory symptoms and airflow limitation. Acute exacerbations may cause respiratory failure and, in severe cases, cardiac arrest. However, structural abnormalities of the upper and central airway may produce clinical features that overlap considerably with asthma.

Structural airway lesions are therefore an important differential diagnosis in patients with difficult-to-treat or atypical asthma. Upper-airway tumours and other laryngeal or subglottic lesions have previously been reported to present as refractory or near-fatal asthma [1-3].

This case talks about a presumed asthma exacerbation that presented as cardiac arrest. It reveals an obstructing supraglottic mass that was missed during initial intubation, probably due to anatomical obscuration, emphasising the importance of reconsidering the anatomical level of airflow obstruction when a presumed asthma exacerbation follows an atypical clinical course.

## Case presentation

The patient was a 74-year-old man with a background of type 2 diabetes mellitus requiring insulin, asthma, hypertension, diabetic nephropathy, previous total knee replacements, and previous spinal fixation. He was independent at baseline. During the preceding year, he had experienced approximately four hospital admissions attributed to asthma exacerbations.

The initial working diagnosis was a life-threatening asthma exacerbation precipitated by a respiratory infection. Despite receiving high-flow oxygen at 15 L/min, his oxygen saturation fell to approximately 50%. Shortly after arrival in the emergency department, he developed pulseless electrical activity (PEA) cardiac arrest. Return of spontaneous circulation was achieved after approximately five minutes and two cycles of cardiopulmonary resuscitation. The arrest was considered secondary to severe hypoxic respiratory failure.

During resuscitation, the patient was intubated using direct laryngoscopy, with no technical difficulty or airway abnormality reported by the emergency department team. Following intubation, auscultation demonstrated only minimal wheeze. Arterial blood gas analysis showed a pH of 7.512 (reference range: 7.35-7.45), partial pressure of oxygen (PaO₂) of 14 kPa (reference range: approximately 10.0-13.3 kPa on room air), and partial pressure of carbon dioxide (PaCO₂) of 4.8 kPa (reference range: approximately 4.7-6.0 kPa). Following return of spontaneous circulation, he was stabilised and transferred to the intensive care unit (ICU) for mechanical ventilation and post-cardiac-arrest care. Chest radiography demonstrated clear lung fields, a normal cardiomediastinal contour, and no pneumothorax or pleural effusion, as shown in Figure 1.

**Figure 1:**
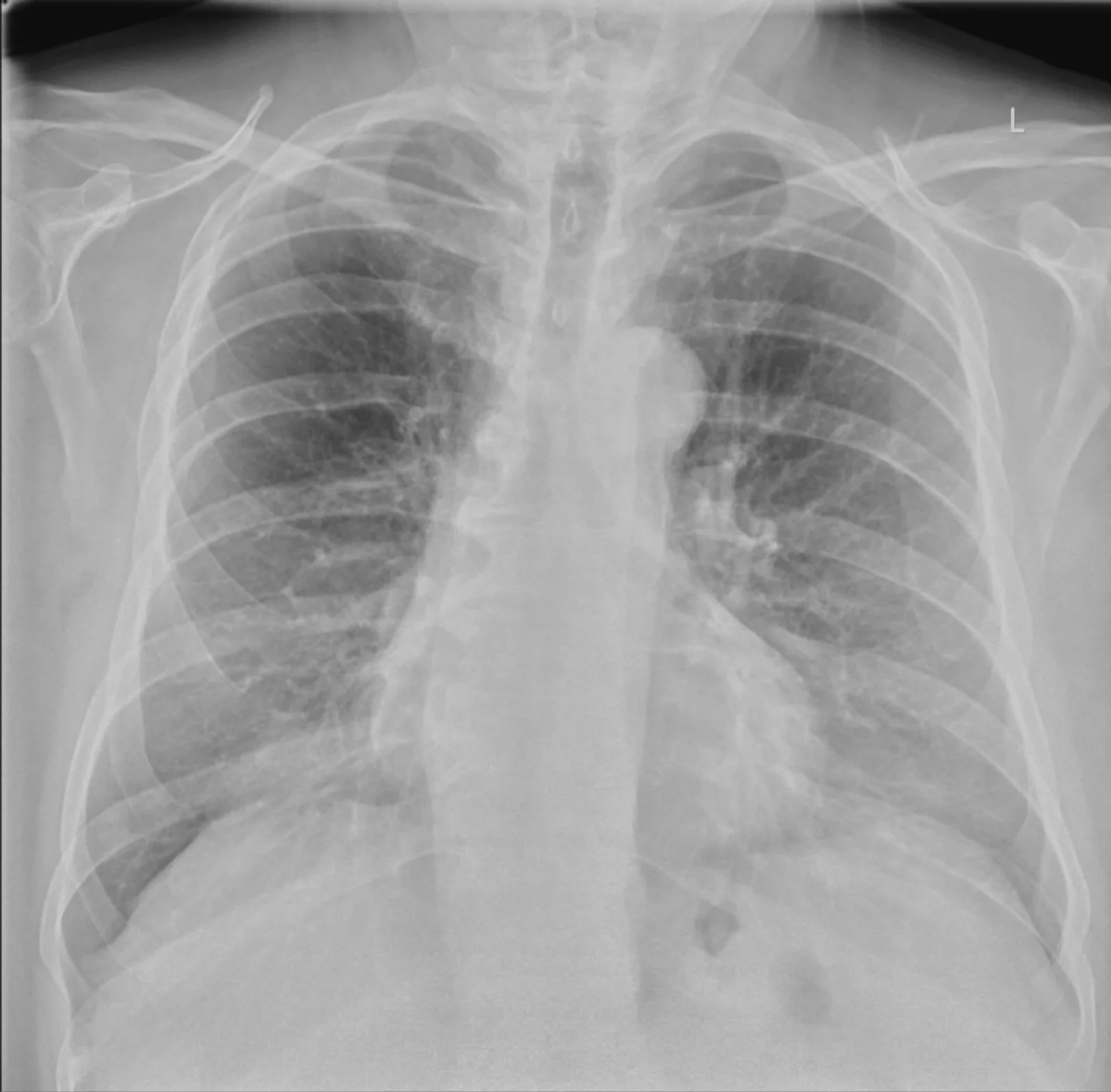
Chest X-ray showing clear lung fields, normal cardiomediastinal contour, and no pneumothorax or pleural effusion

In the ICU, the patient was mechanically ventilated using pressure-regulated volume control and maintained on a fraction of inspired oxygen (FiO₂) of 25-30%. Ventilatory parameters included a respiratory rate of 14-18 breaths/min, tidal volume of approximately 490-500 mL, positive end-expiratory pressure (PEEP) of approximately 5 cmH₂O, and peak airway pressure of approximately 15 cmH₂O. Respiratory examination demonstrated clear bilateral breath sounds without added sounds or evidence of bronchospasm. Arterial blood gas analysis demonstrated a pH of 7.47, PaCO₂ of 4.7 kPa, bicarbonate (HCO₃⁻) of 25 mmol/L (reference range: 22-26 mmol/L), and lactate of 0.99 mmol/L (reference range: 0.5 - 2.2 mmol/L), indicating adequate ventilation without hyperlactataemia.

He received nebulised bronchodilator therapy with salbutamol and ipratropium bromide, systemic hydrocortisone, magnesium sulphate, and aminophylline, together with empirical cefepime and doxycycline. Admission blood tests demonstrated leukocytosis, with a white cell count (WCC) of 26.5 × 10⁹/L (reference range: 4 - 11 x 10⁹/L).

Cardiac biomarkers demonstrated an increase in troponin from approximately 10 to 129 ng/L, while B-type natriuretic peptide (BNP) was approximately 1,117 ng/L (reference range: >400 ng/L). Electrocardiography demonstrated fast atrial fibrillation, for which digoxin was commenced.

Given the circumstances surrounding the arrest, cardiology considered the event to be more consistent with a secondary hypoxic cardiac arrest than a primary coronary event. Following assessment of the atrial fibrillation, anticoagulation with apixaban 5 mg orally twice daily was commenced.

The following morning, respiratory examination demonstrated clear bilateral breath sounds without significant wheeze or bronchospasm. A sedation hold was therefore undertaken to assess neurological recovery and readiness for extubation. Within approximately 15 minutes of discontinuing sedation, the patient became markedly agitated, attempting to remove lines and climb out of bed. He demonstrated globally increased tone with limited purposeful response, and sedation was therefore restarted.

Following further stabilisation and reassessment, extubation was attempted later that day.

Immediately following extubation, the patient developed marked inspiratory stridor, severe respiratory distress, and profound hypoxaemia, with oxygen saturation falling rapidly from 100% to approximately 60%. Emergency re-intubation was undertaken using video laryngoscopy. This unexpectedly revealed a large polypoid supraglottic mass adjacent to the vocal cords and cuneiform cartilage. The lesion distorted the laryngeal inlet and appeared to produce dynamic, ball-valve obstruction, as demonstrated in Figure 2.

**Figure 2:**
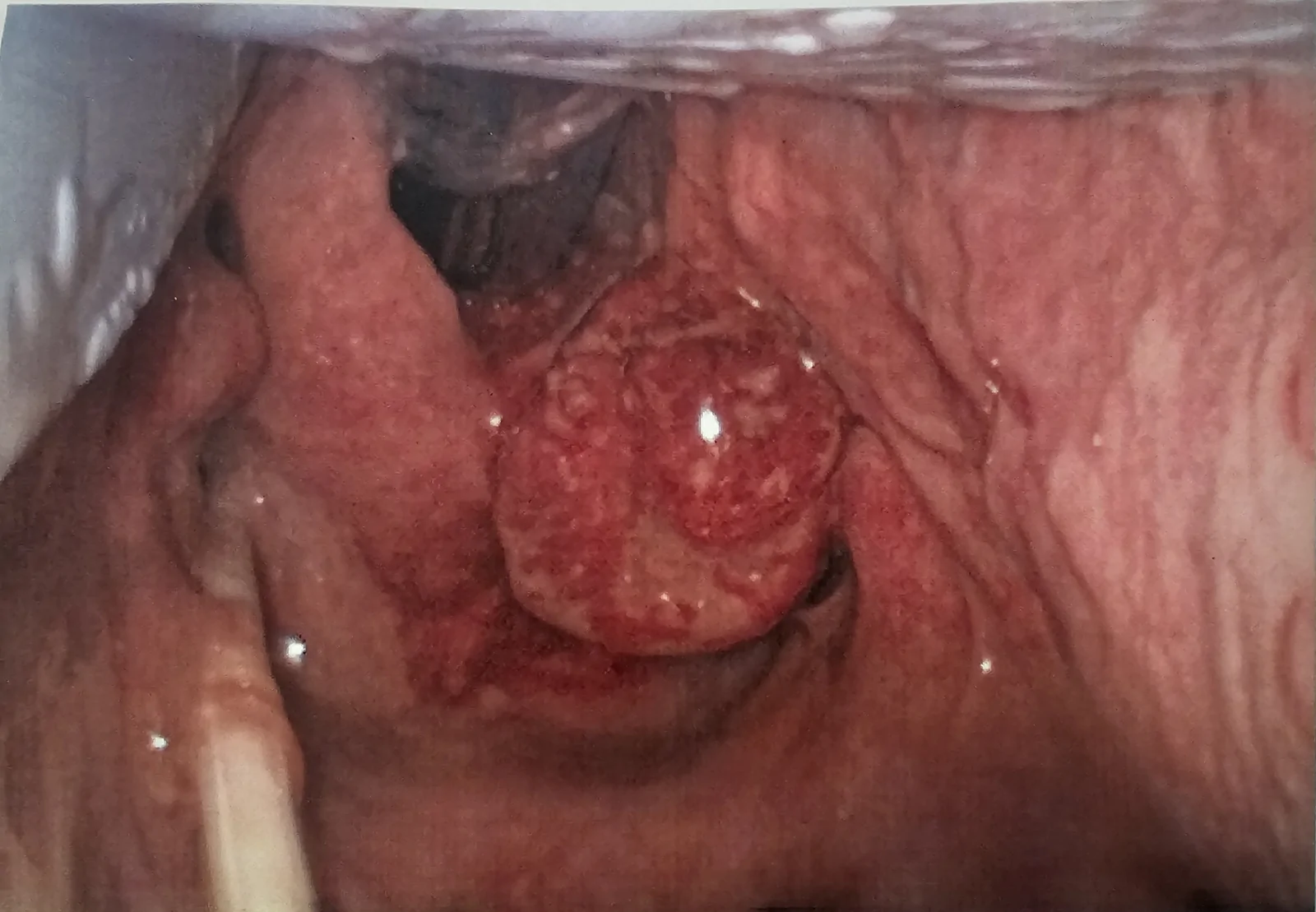
Video-laryngoscopic view demonstrating a large polypoid supraglottic mass with an irregular erythematous surface, partially obstructing the laryngeal inlet

Re-intubation was technically difficult. Following an unsuccessful initial attempt, senior anaesthetic assistance was obtained, and an endotracheal tube was successfully passed over a bougie. Oxygen saturation subsequently recovered rapidly to 100%.

Urgent otorhinolaryngology (ORL) review was obtained. The initial differential diagnosis included a vallecular cyst and malignancy. Further attempts at extubation were therefore avoided pending definitive airway assessment. Contrast-enhanced computed tomography (CT) of the neck, chest, and abdomen demonstrated no convincing neck mass, significant distal extension, or evidence of metastatic disease, despite the lesion having been directly visualised during video laryngoscopy.

On subsequent ORL consultant assessment in the ICU, the lesion was initially difficult to identify because it was concealed within the laryngeal fossa. Using the previous video-laryngoscopy images for anatomical guidance, the lesion was subsequently located, confirming the presence of a right supraglottic mass.

Following multidisciplinary discussion, the patient was taken to the theatre for definitive airway assessment and surgical management. The lesion was completely excised, satisfactory local haemostasis was achieved, and the specimen was sent for histopathological examination.

Following the procedure, the patient remained intubated and sedated pending further airway assessment and was kept nil by mouth. After surgical removal of the lesion, he entered a period of progressive clinical recovery with de-escalation of critical care support.

Inflammatory markers fluctuated during the patient's critical illness and postoperative recovery. The admission white cell count (WCC) of 26.5 × 10⁹/L decreased to 17.1 × 10⁹/L on day 2 and to approximately 10.9 × 10⁹/L on day 3. C-reactive protein (CRP) increased from 2 mg/L on admission to 29 mg/L on day 2, 39 mg/L on day 3, and approximately 50 mg/L on day 4 before decreasing to 11 mg/L by day 5. Procalcitonin remained relatively low and demonstrated a declining trend.

Renal function remained broadly preserved throughout the admission despite the patient's background of diabetic nephropathy. The patient's laboratory investigations are summarised in Table 1.

**Table 1:** Haematological and biochemical parameters during hospital admission White cell count (WCC), C-reactive protein (CRP), B-type natriuretic peptide (BNP)

Investigation	Reference range	Day 1	Day 2	Day 3	Day 4	Day 5	Day 6
Haemoglobin (g/L)	115–160	157	147	130	127	144	152
WCC (×10⁹/L)	4–11	26.5	17.1	14.9	10.7	12.4	10.5
Sodium (mmol/L)	133–146	135	141	142	141	147	143
Potassium (mmol/L)	3.5–5.3	3.8	4.1	4.5	4.4	3.6	4.6
Urea (mmol/L)	2.5–7.8	9.8	8.0	8.4	10.3	9.5	4.8
Creatinine (µmol/L)	45–84	73	86	79	75	61	67
CRP (mg/L)	0–5	2	29	50	39	~20	11
Procalcitonin	Laboratory-specific	0.08	0.29	0.23	0.17	0.14	0.12
Troponin	<14	10.4 → 129	–	–	–	–	–
BNP	Laboratory-specific	1117	–	–	–	–	–

The patient was then extubated, moved to the ward and returned to baseline after two weeks of hospital admission. During follow-up, histopathology results demonstrated granulation tissue. No specific neoplastic diagnosis was identified.

## Discussion

Structural upper-airway lesions are uncommon but important mimics of asthma [1-3]. In this case, there was an absence of significant bronchospasm following intubation and stridor, followed by profound hypoxaemia on extubation. This prompted consideration of an alternative mechanism of airway obstruction. Emergency video laryngoscopy subsequently revealed a large supraglottic mass, making the failed extubation the key diagnostic event.

The lesion was not recognised during the initial emergency intubation and was subsequently difficult to identify during specialist assessment because of its concealed position. Mobile upper-airway lesions may cause dynamic obstruction, including a “ball-valve” mechanism [3,4]. While the patient was intubated, the endotracheal tube effectively bypassed the supraglottic obstruction. Following extubation, the lesion was again able to obstruct the native airway, providing a plausible mechanism for the abrupt development of stridor and profound hypoxaemia and the rapid recovery following re-intubation.

Histopathological examination demonstrated granulation tissue without evidence of malignancy. This finding highlights that benign inflammatory laryngeal lesions can nevertheless produce severe and potentially fatal upper-airway obstruction [5].

Supraglottic lesions, including epiglottic cysts, can cause severe and potentially life-threatening airway obstruction [6]. Structural upper-airway pathology should therefore be reconsidered in patients treated for asthma who develop atypical features, particularly stridor or unexplained severe hypoxaemia.

Cross-sectional imaging is valuable for assessing laryngeal pathology and its relationship with surrounding structures [7]. In this patient, however, CT of the neck did not demonstrate a convincing abnormality despite the lesion having been clearly visualised during emergency video laryngoscopy. This discrepancy illustrates the diagnostic importance of direct airway visualisation when suspicion of structural laryngeal pathology remains high despite inconclusive cross-sectional imaging.

The supraglottic lesion may also have contributed to the severe respiratory deterioration preceding the PEA cardiac arrest. However, direct causality cannot be established because concomitant asthma and respiratory infection remained potential contributing factors. This case emphasises the importance of reconsidering structural upper-airway obstruction when presumed asthma follows an atypical clinical course, particularly when stridor, failed extubation, or otherwise unexplained severe hypoxaemia occurs.

## Conclusions

Structural upper-airway lesions are uncommon but potentially life-threatening mimics or coexisting causes of respiratory deterioration in patients with asthma. In this case, persistent severe hypoxaemia and stridor following extubation, despite improvement in lower-airway bronchospasm, led to the identification of a previously unsuspected mobile supraglottic lesion causing dynamic airway obstruction. Re-intubation beyond the obstruction rapidly restored oxygenation, and subsequent excision demonstrated benign granulation tissue on histopathological examination.

This case highlights the importance of considering structural upper-airway obstruction when the clinical course is atypical for asthma, particularly when stridor or unexpected deterioration occurs following extubation. It also demonstrates that benign histopathology does not necessarily imply benign physiological behaviour. Within the confined supraglottic airway, the location and mobility of a lesion may be sufficient to produce life-threatening obstruction.
